# Histotripsy: A Method for Mechanical Tissue Ablation with Ultrasound

**DOI:** 10.1146/annurev-bioeng-073123-022334

**Published:** 2024-06-20

**Authors:** Zhen Xu, Tatiana D. Khokhlova, Clifford S. Cho, Vera A. Khokhlova

**Affiliations:** 1 Department of Biomedical Engineering, University of Michigan, Ann Arbor, Michigan, USA; 2 Applied Physics Laboratory, University of Washington, Seattle, Washington, USA; 3 Department of Surgery, University of Michigan, Ann Arbor, Michigan, USA; 4 Department of Acoustics, Lomonosov Moscow State University, Moscow, Russia

**Keywords:** focused ultrasound surgery, high-intensity focused ultrasound, HIFU, nonlinear waves, cavitation, boiling, mechanical bioeffects, histotripsy

## Abstract

Histotripsy is a relatively new therapeutic ultrasound technology to mechanically liquefy tissue into subcellular debris using high-amplitude focused ultrasound pulses. In contrast to conventional high-intensity focused ultrasound thermal therapy, histotripsy has specific clinical advantages: the capacity for real-time monitoring using ultrasound imaging, diminished heat sink effects resulting in lesions with sharp margins, effective removal of the treated tissue, a tissue-selective feature to preserve crucial structures, and immunostimulation. The technology is being evaluated in small and large animal models for treating cancer, thrombosis, hematomas, abscesses, and biofilms; enhancing tumor-specific immune response; and neurological applications. Histotripsy has been recently approved by the US Food and Drug Administration to treat liver tumors, with clinical trials undertaken for benign prostatic hyperplasia and renal tumors. This review outlines the physical principles of various types of histotripsy; presents major parameters of the technology and corresponding hardware and software, imaging methods, and bioeffects; and discusses the most promising preclinical and clinical applications.

## INTRODUCTION

High-intensity focused ultrasound (HIFU) is a nonionizing, noninvasive therapy that uses ultrasound beams focused from outside of the body toward a targeted site to locally induce a desired bioeffect (1). Most current clinical HIFU applications rely on the thermal effect of long ultrasound bursts, which rapidly heat and generate localized thermal ablation of tissue in the focal region of a HIFU beam (2). HIFU has been approved by the US Food and Drug Administration (FDA) to treat multiple diseases, including uterine fibroids (3), prostate tumors (4), and essential tremor (5).

Histotripsy is a relatively new HIFU-based technology that aims not to heat but to mechanically liquefy targeted tissue into subcellular debris using sequences of short, high-amplitude focused ultrasound pulses, which generate bubble activity at the focus (6). In contrast to conventional HIFU thermal therapy, the major mechanism of histotripsy is mechanical, which enables localized tissue disintegration at the focus without thermal damage to the overlaying and surrounding tissues. Since histotripsy was introduced in 2004 (7), various approaches, the required acoustic field parameters, the underlying physical mechanisms, and induced bioeffects have been extensively explored, and histotripsy is finding use in various clinical applications (8, 9).

**Figure 1:**
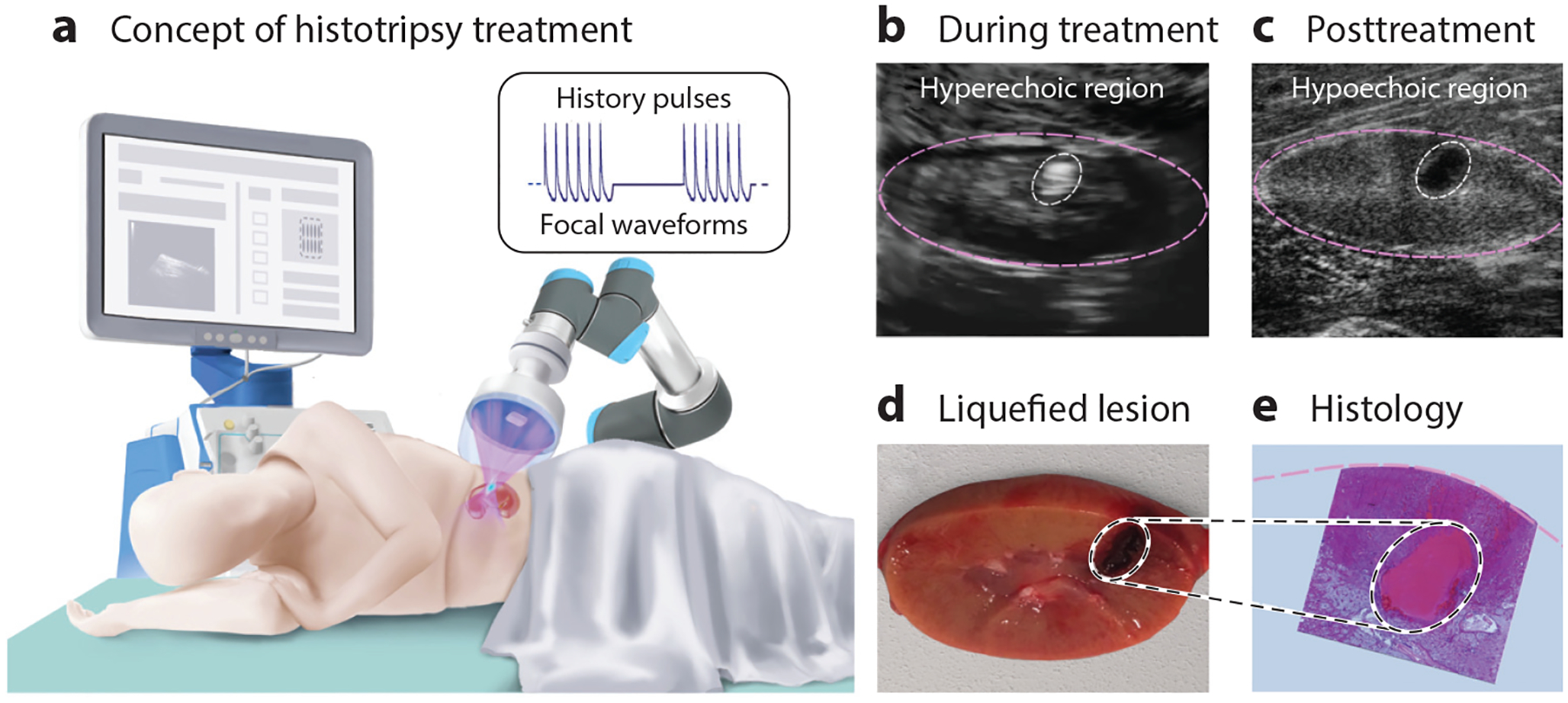
Schematic illustration of histotripsy treatment. (*a*) A pulsed beam is focused through the skin and translated either mechanically or electronically over the target volume to liquefy tissue. (*b*) Sonication is visualized in real time as an echogenic region due to the presence of bubbles. (*c*) The posttreatment region is hypoechogenic due to the loss of scatterers. (*d*) The shape and dimensions of the liquefied lesion agree well with brightness-mode visualization and (*e*) histology.

Figure 1 illustrates the general concept of histotripsy. A diagram of kidney tissue is used as an example. A high-intensity pulsed ultrasound beam is focused noninvasively, through the skin, to the targeted site. Short pulses with a duration ranging from microseconds to milliseconds are delivered to the focus at a low duty cycle to generate gas and vapor bubbles via cavitation or rapid boiling (Figure 1*a*). The bubble activity results in mechanical disintegration or liquefaction of tissue. Histotripsy can be monitored in real time using conventional brightness-mode (B-mode) ultrasound, because bubbles are strong scatterers and appear hyperechoic on ultrasound images (10–12) (Figure 1*b*). The progression of the treatment can also be monitored on B-mode ultrasound because fractionation of tissue destroys scatterers, thereby producing a hypoechoic cavity, and the degree of hypoechogenicity corresponds to the degree of tissue liquefaction (13–15) (Figure 1*c*). To obtain a volumetric lesion of clinically relevant size, the transducer focus can be moved throughout the targeted volume, either mechanically or electronically using a multielement phased array (14, 16–18). Finally, a liquefied lesion with sharp margins from completely destroyed to completely intact tissue forms at the focus (Figure 1*d*,*e*). The shape and dimensions of the lesion agree well on B-mode, gross sections, and histology (14, 17).

A histotripsy approach therefore has specific clinical advantages compared with thermal HIFU: the capacity for real-time treatment monitoring using ultrasound imaging due to the presence of bubbles and evaluation of the treatment outcomes due to the loss of tissue structures in the liquefied lesion. Another important characteristic of histotripsy is tissue selectivity: Connective tissue structures (e.g., blood vessels, biliary structures) are more resistant to mechanical ablation than are cells (17, 19, 20). The tissue selectivity allows treatment of the target tumors while preserving critical structures such as major vessels and bile ducts. Furthermore, the nonthermal mechanism of the approach results in a sharper boundary and higher treatment precision compared with thermal ablation, which is limited by heat sinking and heat diffusion effects. Because of the binary nature of the bubble activity, histotripsy generates precise lesions with a sharp transition from completely lysed to intact tissue. Histotripsy-treated liquefied tissue is reabsorbed by the body over 1–2 months, resulting in effective removal of the treatment volume. Only millimeter-sized scar tissue remains, which does not interfere with future tumor screening at the treatment site as thermally denatured tissue would.

Two major approaches—histotripsy, sometimes termed cavitational histotripsy (CH), and boiling histotripsy (BH), with two different physical mechanisms—have recently been extensively explored (21). To differentiate the different types of histotripsy, the terms CH and BH are used throughout this review. CH relies on initiation of a dense bubble cloud using microsecond-long pulses (22). By repeatedly expanding and collapsing during each pulse, the cavitation cloud completely homogenizes the tissue (23). CH has been realized in many organs ex vivo and in vivo, including prostate (24, 25), heart (10, 26), liver (12, 17), kidney (27, 28), brain (29, 30), thyroid (31), and pancreas (32), as well as blood clots (33, 34). BH uses milliseconds-long pulses containing shock fronts to rapidly heat tissue to boiling temperature and produce a vapor bubble at the focus within each pulse (35). The interaction between the rest of the pulse and the vapor cavity results in mechanical fractionation of tissue (36, 37). BH has been performed in ex vivo tissues and in vivo in liver (11, 14), kidney (14), prostate (38), large hematomas, and abscesses (39). In addition, a hybrid histotripsy (HH) approach that uses submillisecond pulses (intermediate between BH and CH) has been proposed and successfully tested in ex vivo porcine liver, kidney, and cardiac muscle (40).

Currently, histotripsy therapy is being evaluated in preclinical studies with small and large animal models for treating cancer (28, 41, 42), cardiac diseases (43, 44), thrombosis (33, 34), hematomas (45, 46), and abscesses (39); enhancing tumor-specific immune response (32, 47, 48); and neurological applications (29, 30, 49). The first clinical trials using CH for benign prostatic hyperplasia, liver cancer, and renal cancer have been undertaken (50, 51). Histotripsy was recently approved by the FDA to treat liver tumors. In addition to complete tissue liquefaction, a mechanism of a partial mechanical tissue damage (not yet histotripsy) is being explored for a broader range of applications such as noninvasive liquid biopsy (52), enhanced drug delivery (53), treatment of muscular-skeletal diseases (54), and biofabrication (20).

Even though the field of histotripsy has significantly matured during the last decade, many aspects remain intriguing and are yet to be studied. It is an exciting and rapidly growing area of research that includes fundamental physics, nonlinear acoustics, cavitation, wave propagation and interaction with multiphase media, mechanical tissue properties, and physiological responses of various organs to localized tissue and cell destruction. This review outlines the current state of the field; physical principles of various types of histotripsy; major parameters of the technology and corresponding hardware and software, imaging methods, and bioeffects; and promising preclinical and clinical applications.

## PHYSICAL MECHANISMS AND ULTRASOUND FIELD PARAMETERS

Histotripsy relies on controlled generation of bubble activity by sequences of ultrasound pulses delivered at a low repetition rate to mechanically disrupt the targeted tissue. With a sufficient number of pulses, the target tissue is liquefied to acellular debris, with no intact cells inside the target volume. The mechanisms underlying histotripsy consist of two parts: generation of bubbles and the tissue homogenization process. Specific differences in such mechanisms are detailed below for three types of histotripsy: CH, BH, and HH. Representative values of the ultrasound field parameters associated with these methods are given in Table 1.

### Cavitational Histotripsy

The CH approach uses nanometer- or micrometer-sized gas pockets inside the tissue as cavitation nuclei to generate a cloud of microbubbles (i.e., a cavitation cloud) (55, 56). CH typically uses microsecond-length pulses (1–20 cycles) at a center frequency of 200 kHz to 5 MHz and relies on very high peak negative pressure in situ (7, 57, 58). There are two CH approaches to generate a cavitation cloud: the intrinsic threshold approach (55) and the shock scattering approach (22).

Using the intrinsic threshold approach, a cavitation cloud can be reliably generated using a single one- to two-cycle ultrasound pulse when the peak negative pressure exceeds the cavitation threshold intrinsic to the tissue. This threshold was measured to be from −26 to −28 MPa in water-based tissue, including blood clots, kidney, liver, heart, and spleen, and to be −14 MPa in lipid-based tissue (55). The intrinsic threshold does not change significantly with transducer frequency, focusing angle (f-number, or f#), or tissue stiffness (59, 60) but reduces with increasing temperature (61). Simulation shows that the pressure level of this threshold corresponds to the surface tension of gas pockets of 2–10 nm in diameter, which are likely the endogenous gas pocket sizes in the tissue (55, 56). These nanometer-sized gas pockets are highly stabilized due to their high surface tension, but when the negative pressure phase of the ultrasound waveform overcomes their surface tension even for a fraction of a microsecond, they can expand rapidly to form cavitation microbubbles. As the ultrasound pulse propagates through the focal zone, cavitation microbubbles are generated within the region where the negative pressure phase is beyond the intrinsic threshold (55, 58). If the peak negative pressure is right above the intrinsic threshold, this cloud of cavitation microbubbles can be smaller than the focal zone. Thus, the intrinsic threshold CH is also termed microtripsy, for its capability of generating a sub-millimeter-sized cavitation cloud smaller than the diffraction limit defined by the ultrasound wavelength (58).

When the peak negative pressure is below the intrinsic threshold, a cavitation cloud can be generated via an indirect approach, termed shock scattering CH (22). Using a multicycle ultrasound pulse (typically 3–20 cycles), an individual microbubble can be generated in the first one to three cycles with a peak negative pressure of approximately −20 MPa in water-based tissue, if there is a preexisting cavitation nucleus (e.g., a 100-nm gas pocket) in the focal zone. At the high pressures used in histotripsy, due to nonlinear acoustic propagation, the shock front develops in each cycle of the ultrasound waveform, and the peak positive pressure in the waveform is much higher than the peak negative pressure (e.g., it can be p+ > 80 MPa, corresponding to | p− | = 20 MPa). The high-amplitude shock in the subsequent acoustic cycle of a multicycle ultrasound pulse scatters back from the soft boundary of the initial individual microbubble and inverts its shape into the shock with a high negative pressure. This inverted shock and the incoming negative phase in the subsequent acoustic cycle together result in extremely high negative pressure, well beyond the intrinsic threshold, and generate a cloud of dense microbubbles. The shock wave in the next acoustic cycle scatters from the newly formed cavitation cloud and repeats the process.

Compared with intrinsic threshold CH (|p− | > 30 MPa), shock scattering CH requires lower peak negative pressure (|p− | ~ 20–30 MPa), and the cavitation cloud formed via shock scattering CH is often denser and larger than the size of the focal zone, resulting in a higher efficiency but lower treatment accuracy compared with intrinsic threshold CH (62). The main downside of shock scattering CH is the stochastic nature of the formation of the first individual bubble, which often requires multiple pulses (from fewer than 10 to more than 100 pulses) to form this bubble to initiate the shock scattering process.

Both intrinsic threshold and shock scattering CH use a very low duty cycle (ultrasound on-time/total treatment time = 0.0001–1%) to mitigate heating. The cavitation bubbles are observed to be generated outside cells in the fluid inside the extracellular matrix (63). After cavitation nucleation, the nanometer-sized gas pockets can grow to hundreds of micrometers and collapse within a few hundred microseconds. The rapid expansion of the cavitation bubbles squeezes the adjacent cells, the bubble collapse pulls the adjacent cells, and the local high strain and stress produced over multiple pulses cause cyclic strain and stress to impose on the cells and eventually mechanically break them down (63). Increasing the number of pulses results in an increase in the extent of cellular and subcellular disruption within the focal zone. Eventually, the tissue within the focal zone can be completely liquefied into acellular debris, with no intact cells remaining. The boundary zone between complete cell disruption and the surrounding intact cells is very narrow, within 1 mm in vivo and 100 μm in vitro. The histotripsy damage threshold is impacted by tissue stiffness (64). In stiffer tissue, the bubble expansion is restricted (62, 65). Stiffer tissue typically also has a higher ultimate tensile stress, which is the maximum stress that a material can withstand while being stretched or pulled before breaking. Thus, stiffer tissue (e.g., tissue with more collagen/fiber components such as tendons, vessels, nerves, or connecting system) has a higher resistance to histotripsy-induced damage; that is, the pressure or the number of pulses required to damage the target tissue is higher with stiffer tissue (64–66). As a result, tissue-selective CH ablation has been observed (12, 17, 67, 68). For example, target liver tissue is completely liquefied to acellular debris while major vessels and bile ducts remain intact inside the liquefied region (17, 68).

### Boiling Histotripsy

The BH approach uses 1- to 10-ms pulses with lower focal pressures and, similar to shock scattering CH, relies on nonlinear propagation effects and the presence of shock fronts in the ultrasound pressure waveform at the focus (69, 70). As ultrasound absorption increases with frequency, tissue heating is significantly enhanced, in tens of times, due to absorption at the shock fronts, which are formed by high harmonics of the initially harmonic wave (71). While in harmonic wave fields the absorption of ultrasound energy and the corresponding heating rates are proportional to the wave intensity or pressure amplitude squared, the absorption at the shocks is proportional to the shock amplitude сubed, and thus grows rapidly with increases in the shock amplitude (35, 71).

Due to the enhanced tissue heating through absorption at the shock fronts, the tissue at the focus can reach a temperature of 100°C within a few milliseconds, resulting in initiation of highly localized boiling bubbles, which explosively grow, cool down, and form a millimeter-scale vapor bubble (35). The interaction of the incoming shocks in the remainder of the pulse with the tissue–vapor interface results in the formation of an acoustic fountain directed into the vapor cavity and consisting of micrometer-scale fragments of ejected tissue—a process known as acoustic atomization (36). At the same time, reflection of shocks from the vapor bubble interface initiates violent prefocal cavitation in tissue immediately proximal to the bubble surface, thus aiding the atomization and microjetting processes (35, 72). The tissue particles are ejected from the proximal bubble wall at velocities that can reach meters per second, and when this jet impacts the distal side of the bubble, it produces additional mechanical damage (45). The abovementioned processes are repeated within each pulse, leading to the formation of a small oblong lesion first, which gradually enlarges and then transforms into a tadpole-like shape. The lesion size saturates within the dimensions of the focal lobe after 30–120 pulses are delivered, depending on the tissue stiffness. Interestingly, thermal effects are negligible within BH lesions because the shocks are superfocused, and the shock-heated region is very small (~100 μm wide and 1 mm long), much smaller in volume than the focal region of the linearly focused beam (36, 71).

To produce purely mechanical lesions, BH typically operates at duty cycles of 0.5–2%. Compared with CH, a relatively higher duty cycle can be used for pulses, and interpulse periods are longer, allowing heat to diffuse and mitigating its accumulation. The peak negative focal pressure (|p− | ~ 10–18 MPa) and overall acoustic output power required for BH are lower than those for CH, but to attain boiling within 1 to 10 ms, shock fronts with amplitudes from 50 to 100 MPa and frequencies higher than 1 MHz are required. The shock amplitudes can be controlled by the transducer focusing angle, which is higher for more focused transducers but can be f# = 1 using conventional HIFU systems. The BH lesion shape also depends on the focusing angle, which is more rounded with a shorter tail for highly focused fields. The size of BH lesions is inversely proportional to frequency, with frequencies higher than 3.5 MHz producing very small lesions. Similar to CH, BH produces precise mechanical ablation with a sharp transition from completely lysed to intact tissue occurring within 100 μm in the absence of tissue motion. It also has tissue-selectivity capabilities, like CH, and thus can safely ablate tissue near critical structures (37). Operating with higher shock amplitudes allows for the use of shorter pulses (e.g., 1 ms versus 10 ms) to generate boiling that accelerates BH treatments (73).

### Hybrid Histotripsy

Most recently, HH that combines CH and BH has been explored (74, 75). HH uses pulse durations of hundreds of microseconds and pressure levels between those of CH and BH (40). Dual-frequency and two-stage approaches have been investigated for HH to enhance the treatment speed (74, 75). HH is in its infancy, and the effective parameter range and precise mechanism need to be fully studied.

## INSTRUMENTATION

Major parts of complete histotripsy systems, similarly to conventional thermal HIFU, include a high-power transducer to generate ultrasound, a high-power electronic driver to feed the transducer, a positioner to move the transducer, an acoustic coupler to ensure efficient transmission of ultrasound into tissue, and imaging to guide and monitor the treatment. Due to the distinct mechanisms of mechanical disintegration of tissue by ultrasound, certain parameters of this instrumentation are specific for histotripsy.

Histotripsy treatments generally require the use of significantly higher peak acoustic power outputs from a transducer compared with conventional thermal HIFU to achieve the desired peak pressures at the focus (70). While peak power ranges from a few hundred watts to tens of kilowatts, the time-average power due to the low duty cycle of less than 1% is relatively low. Intrinsic threshold CH is realized with the shortest pulses, the highest focal pressures, and, thus, the highest peak power. For other types of histotripsy, characteristic peak power and focal pressure requirements decrease with an increase of the pulse length; that is, they are lower for shock scattering CH and then for HH, and are the lowest for BH (Table 1). To comply with the power and focal pressure requirements for histotripsy, specialized driving electronics systems and transducers have been developed.

**Figure 2:**
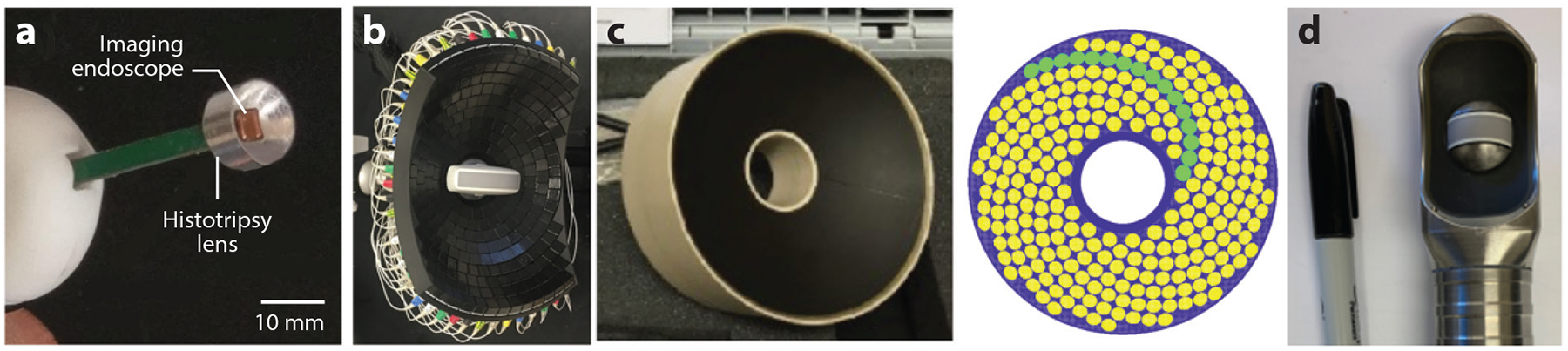
Representative histotripsy transducers. (*a*) A 6.8-MHz, 10-mm shock scattering cavitational histotripsy (CH) transducer with a 4-mm square hole in the center for a high-frequency imaging probe (79). (*b*) A 750-kHz truncated circular aperture array transducer (165 × 234-mm aperture and 142-mm focal distance) for intrinsic threshold CH in liver and kidney (77). (*c*) A 1.5-MHz, 256-element array (144-mm aperture and 120-mm focal distance) for boiling histotripsy and hybrid histotripsy (18). (*d*) A 2-MHz transrectal transducer for boiling histotripsy of prostate tissue.

CH uses microsecond-long pulses with extremely high pressure. To generate such short pulses, the ultrasound transducers need to have a wide bandwidth. To generate high pressure, relatively large transducers with a low f# (transducer diameter/focal distance < 1) are typically used (76, 77). The transducer geometry varies from a spherical segment for brain to a truncated circular aperture shape for liver and kidney to better fit an acoustic window available for the treatment (76, 77). More recently, millimeter- to centimeter-sized small transducers have been developed for endocavity or endoscopic applications of histotripsy (78–80) (Figure 2*a*). These transducers often have higher frequency (>3 MHz) to achieve a higher focal gain and can be integrated with a high-frequency imaging probe to guide the treatment (79), although a lower-frequency (1-MHz) endocavity transducer was recently developed to enable larger-volume treatment (80). The electronic driver provides short bursts of 1–3 kV, a much higher voltage compared with thermal HIFU, BH, and HH that cannot be generated by a commercial linear amplifier. A current-based amplifier and a half-bridge class-D amplifier have been designed and used for intrinsic threshold CH and shock scattering CH, respectively (77, 81). Most recently, a transmit-and-receive-capable electronic driver has been developed for CH to enable aberration correction (82) and acoustic cavitation mapping (83).

An important step in accelerating histotripsy studies was the development of rapid prototyping of custom transducers using modular piezoelectric elements and 3D-printed housing (84). This approach enabled multiparametric studies exploring the effects of transducer frequency, shape, and focusing geometry for various histotripsy methods and applications (59, 76, 85). Recent developments include rapid prototyping of multielement phased arrays for microtripsy designed to enable electronic focus steering and aberration correction (77). The arrays have been constructed either with monolithic-shaped housing to encase the modular piezoelectric elements (76) or by combining separate individual elements with a thin insulation layer that permitted modification of the array’s shape and easier replacement of broken elements (77). For example, a 750-kHz, 260-element truncated circular array (165 × 234-mm aperture and 142-mm focal distance) was designed and built for histotripsy liver treatment (Figure 2*b*). A 700-kHz, 360-element hemispherical array (150-mm focal distance) was designed and built for transcranial histotripsy brain treatment. Both arrays are driven with transmit-and-receive-capable electronics, such that aberration correction (82, 86) and acoustic cavitation mapping can be implemented (83).

A high-voltage electronic pulser originally developed for CH was further enhanced for use with longer pulses up to 10 ms in BH (85). A combination of this driving system with various transducers built using rapid prototyping was applied for investigating BH mechanisms and developing systems for various clinical applications (36, 39, 45, 87).

While CH cannot be realized using commercially available transducers and power amplifiers, both HH and BH can be implemented using commercial amplifiers (35, 71), as well as the existing clinical and preclinical HIFU systems with ultrasound or magnetic resonance (MR) guidance. The ultrasound-guided VIFU 2000 preclinical system (Alpinion Medical Systems, Bothell, Washington, USA) operating with a 1.5-MHz single-element transducer (64-mm aperture and 45-mm radius of curvature) has been used in small animal BH studies at peak electrical power levels from 200 to 600 W (48, 52, 88). An ultrasound-guided transmit-and-receive-capable BH system has been developed based on the power-enhanced 256-channel Verasonics ultrasound engine (3.5-kW max electric power) and a custom-designed piezocomposite 1.5-MHz spiral phased array (144-mm aperture and 120-mm radius of curvature) (Figure 2*c*). The system enabled trans-abdominal BH ablation and aberration correction in in vivo porcine liver and kidney (18, 89). A miniaturized 2-MHz (Figure 2*d*) transducer was recently developed for endorectal ultrasound-guided BH ablation of prostate and was successfully tested in in vivo canine studies (90).

The clinical MR-HIFU Sonalleve V1 and V2 systems (Profound Medical Inc., Mississauga, Ontario, Canada), which include 256-element, 1.2-MHz phased transducer arrays (127.8-mm and 135.9-mm apertures and 120-mm and 140-mm radii of curvature, respectively), are capable of operating under shock-forming conditions and have been used with peak electrical power ranging from 400 W in BH to 1,000 W in HH experiments (73, 91, 92). A higher-frequency preclinical MR-guided system (Image Guided Therapy, Pessac, France) with a 3-MHz annular phased transducer array (48-mm diameter and adjustable focus depth of 30–80 mm) was used to treat subcutaneous tumors in mice at 350-W electrical power (93, 94).

Most histotripsy transducers have a central opening for holding an ultrasound imaging probe or a cavitation detection probe, such that the imaging plane is aligned with the therapy focus. The presence of the central opening also protects high-power transducers from mechanical damage that most often occurs at their center due to the concentration of Lamb waves at the transducer surface and reflections from tissue interfaces located approximately halfway to the focus.

Acoustic coupling of transducers to tissue is an important component to ensure efficient ultrasound transmission from the transducer to the skin. Acoustic coupling has been done by immersing transducers in a water container with a thin acoustically transparent membrane at the bottom (9, 14, 18, 95), using cone-shaped holders attached to the transducer with a membrane at the end opening and filled with degassed and deionized water (11) or having the transducer stationary in liquid with a membrane and using gel pads (94). In addition, a recent study has shown that a flat aperture Fresnel lens–based CH transducer can eliminate water coupling and use gel instead to allow ultrasound transmission directly to the skin from the transducer surface (96).

To generate volumetric lesions, a positioning system or a robotic arm (9, 14, 17) has been used to mechanically translate a transducer over a set of discrete foci, in combination with electronic focus steering when phased arrays are employed (16, 18, 20, 29). A robotic arm can also be used for breathing motion compensation (89).

Numerical simulation tools and acoustic measurement instrumentation are key components in designing histotripsy transducers and in planning treatments. Initial design-stage simulations help optimize the size and shape of the transducer to ensure its ability to generate the required pressures and/or shocks at the focus. The transducer pressure field is measured in water using a hydrophone. Histotripsy fields are strongly focused, and shocks are highly localized within the focal region; therefore, the hydrophone has to be miniature, broadband, and robust. Fiber-optic hydrophones are commonly used, as they can withstand high pressures, have small fiber tip sizes (~100 μm), and have broad bandwidth (~100 MHz) (73, 85, 97). As an alternative, robust membrane hydrophones comprising a steel foil for front protection from cavitation damage have been proposed for such measurements (98). When planning treatments, in situ focal pressures are predicted from measurements in water by scaling the transducer power to compensate for attenuation losses in tissue (84, 91). To accurately predict full 3D acoustic fields in water and tissue, nonlinear numerical simulations are performed, requiring a realistic pattern of the transducer vibration (18, 91). This pattern can be obtained via acoustic holography, in which low pressure is measured with a hydrophone in water over a plane transverse to the transducer axis and then numerically backpropagated to the transducer surface (91).

Freely available software tools for numerical modeling of histotripsy fields with shocks have been developed (99). Currently, such software is available only for the axially symmetric field geometries. However, transducers generating quasi-axially symmetric fields can be approximated by a model of axially symmetric equivalent source, which yielded good agreement with more robust 3D modeling at a fraction of the computational cost (69, 100).

## IMAGE-GUIDED TARGETING AND MONITORING

**Figure 3:**
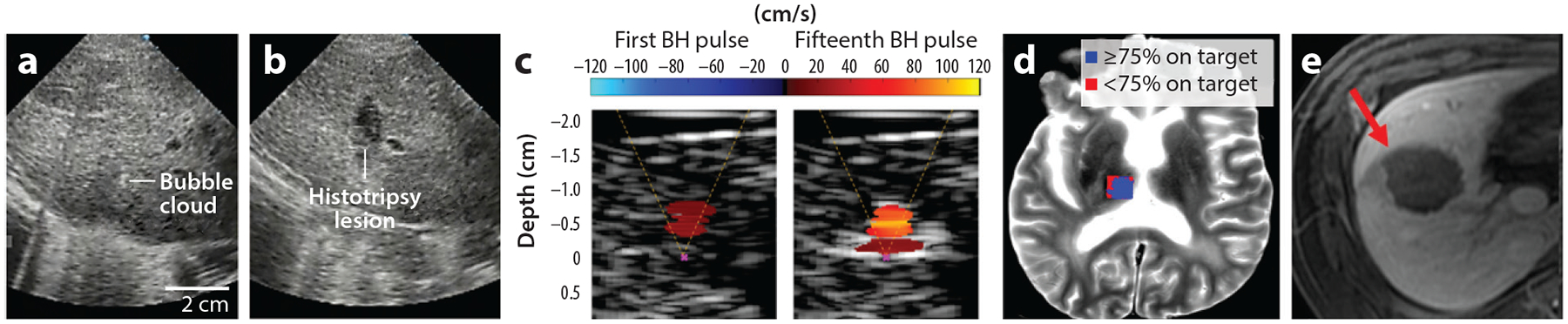
Imaging guidance. (*a*,*b*) Ultrasound brightness-mode images of in vivo porcine liver during and after shock scattering cavitational histotripsy (CH) treatment (103). A hyperechoic-zone cavitation bubble cloud was seen during treatment (*a*), and a hypoechoic region of the histotripsy ablation zone was seen after treatment (*b*). (*c*) Ultrasound color Doppler images in myocardium tissue treated by boiling histotripsy (BH) taken immediately after the first and fifteenth pulses (78). (*d*) Acoustic cavitation map generated by a transmit-and-receive-capable transcranial histotripsy array overlaid on a T2-weighted magnetic resonance (MR) image of a human cadaveric brain. (*e*) Axial postcontrast portal venous phase MR image demonstrating an ablation zone within the right hepatic lobe (*red arrow*) of in vivo porcine liver after shock scattering CH treatment (68).

For a noninvasive ablation, real-time imaging-guided targeting and monitoring are important to ensure high treatment accuracy. HIFU thermal treatment uses magnetic resonance imaging (MRI) thermometry as real-time guidance; however, this requires a few hours of dedicated MRI scan time (101), which is associated with high cost and could be difficult to arrange in hospitals with limited MRI availability. One advantage of histotripsy is that it can be guided by ultrasound imaging (11, 14, 17, 43). Typically, the histotripsy ultrasound transducer has a central hole to house an ultrasound imager that can view the focal plane of the treatment. Histotripsy-generated bubbles are readily visualized as a temporally changing, hyperechoic (bright) zone on standard B-mode ultrasound images (102, 103) (Figure 3*a*). The interference from the histotripsy ultrasound pulses on ultrasound images can be mitigated by synchronizing with imaging ultrasound pulses. As histotripsy mechanically disrupts the cells and tissue structures, which reduces the number and size of sound scattering in the treatment region, the histotripsy treatment zone appears hypoechoic (dark) compared with the untreated region (13, 15, 103) (Figure 3*b*).

As ultrasound B-mode imaging is a standard clinical imaging tool that is widely available at low cost and can be easily incorporated for treatment guidance, it has been used in almost all histotripsy preclinical in vivo studies and clinical trials (33, 43, 50, 51, 104). However, there are limitations of the ultrasound B-mode imaging guidance, as it is only 2D and cannot provide 3D volume information, it cannot clearly visualize some tumors, and it provides only a qualitative assessment of the degree of tissue liquefaction. Beyond the B-mode modality, some other ultrasound imaging methods have been evaluated for histotripsy monitoring. For example, as histotripsy-treated tissue gradually becomes softer and eventually is liquefied, ultrasound elastography can be used to measure the decreasing elastic modulus during the treatment as a quantitative measure of the tissue damage (105). Technically, ultrasound elastography cannot be implemented during histotripsy, and the treatment has to be halted for the measurement. Doppler-based methods have been recently developed and proposed as a quantitative tool to characterize tissue movement or streaming effects in liquefied lesions at the focus and thus to determine the degree of tissue disintegration (78) (Figure 3*c*). Acoustic cavitation emission signals received by either an imaging array for passive cavitation detection/mapping (106) or a histotripsy array with transmit-and-receive capabilities (83) have been used to form 3D cavitation mapping to monitor the treatment (Figure 3*d*). Features can be extracted from the cavitation emission signals to quantitatively correlate with the level of tissue damage; for example, the collapse time increases with an increasing level of histotripsy damage (107). In addition, fast-frame plane-wave ultrasound imaging has been used to monitor bubble dissolution following the collapse of histotripsy cavitation bubbles (108).

MRI and computed tomography (CT) are used clinically for routine tumor screening and treatment follow-up. Both modalities have been used in in vivo histotripsy preclinical studies and clinical trials for immediate posttreatment evaluation and for longitudinal follow-up (12, 20, 41, 102). Contrast-enhanced MRI or CT can be used to differentiate any residual tumors from the histotripsy ablation zones (109). For the brain, where ultrasound imaging is not practical due to the presence of the skull, transcranial histotripsy can be guided by MRI (29, 76). In vivo histotripsy ablation zones can be visualized with T2- or T2*-weighted MRI due to bleeding into the treatment region (12, 41, 102) (Figure 3*e*). Histotripsy ablation zones can also be viewed on MRI with diffusion-weighted imaging (DWI) in vivo and ex vivo, as histotripsy homogenization of cellular matter increases diffusion in the treatment region compared with the surrounding tissues (110). Pretreatment targeting can be achieved by visualizing the focal zone with MR thermometry or MR-ARFI (acoustic radiation force imaging), although this requires the histotripsy transducer to generate longer ultrasound pulses or a higher duty cycle to cause low heating or displacement at the focus. This may be readily achievable with the BH system but may require modification of the CH system. The MR-ARFI method for determining the threshold for shock-forming conditions at the focus was recently used in in vivo animal studies using a clinical MR-HIFU system (111). There is early work showing that histotripsy-induced cavitation can cause local motion detectable using DWI with MRI pulse sequences synchronized with histotripsy pulses, which could provide monitoring during treatment (112), but further investigation is warranted. In BH, the appearance of vapor bubbles at the focus during treatments was detected on MR images using a gradient-echo sequence and confirmed by visual observations in optically transparent polyacrylamide gel phantoms (113).

## THERAPEUTIC APPLICATIONS

As a platform technology, histotripsy has been investigated for many disease types in preclinical research, which are summarized in the following subsections. There have been clinical trials of histotripsy for treatment of benign prostatic hyperplasia and liver cancer, as reported in the section titled Clinical Trials. Histotripsy was recently approved by the FDA for the treatment of liver tumors.

### Cancer Applications

**Figure 4:**
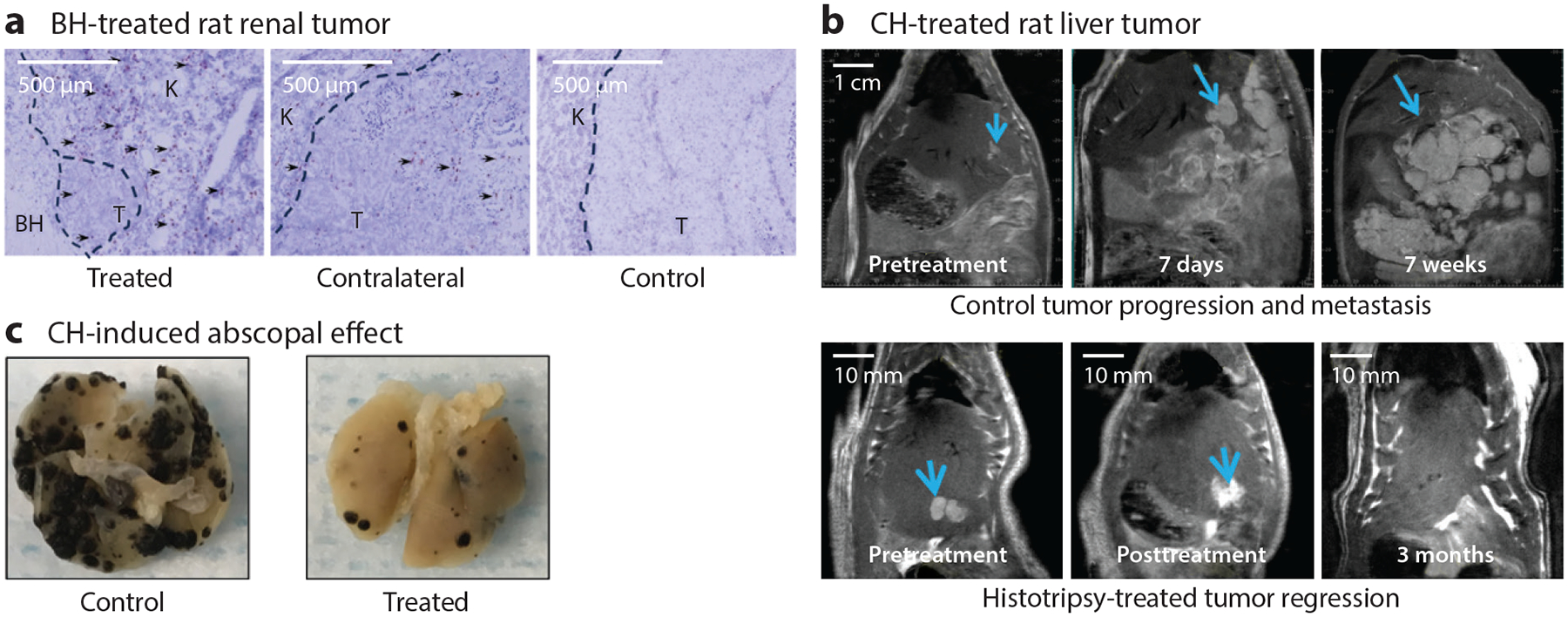
Histotripsy cancer treatment and immunostimulation. (*a*) Immunohistochemistry staining for CD8^+^ T cells (*brown color*, *arrowheads*) of kidney tumor in spontaneous bilateral Eker rat model 48 h following boiling histotripsy (BH) ablation of 50% of a tumor (K = normal kidney; T = tumor). Enhanced CD8^+^ T cell infiltration was observed not only in the residual treated tumor and adjacent kidney tissue but also in the contralateral nontreated tumor and not in untreated controls. (*b*) A rodent McA-RH7777 liver tumor model with spontaneous intrahepatic metastasis and intact immune system in an untreated control case (*top row*) and a histotripsy-treated case (*bottom row*) (42). Partial treatment (50–75% of the tumor volume) using microtripsy led to complete tumor regression and reduced risk of metastasis development. (*c*) Microtripsy cavitational histotripsy (CH) ablation of B16F10 melanoma flank tumors caused abscopal reduction in the number and size of distant, nonablated pulmonary metastases (*black lesions*) (47). Panel *a* adapted from Reference 48.

The ability to liquefy soft tissues in a noninvasive and precisely guided manner makes histotripsy a uniquely attractive approach for oncological therapy. Conventional tumor-directed therapies such as radiation and thermal ablation can be limited by off-target toxicities or the need for direct tumor puncture. A number of in vitro and in vivo preclinical studies have demonstrated the ability of histotripsy to ablate a wide range of histological tumor types, including hepatocellular carcinoma (41, 42), prostate cancer (114, 115), melanoma (47), neuroblastoma, cholangiocarcinoma (116), renal cell carcinoma (28, 48), pancreatic adenocarcinoma (32, 88) (Figure 4*a*), osteosarcoma (117), and glioma (109). Slowed tumor growth, increased survival, or even reduced metastasis (abscopal effects) following complete or partial histotripsy tumor ablation have been reported. For example, Worlikar et al. (42) showed that 9 of 11 rats with McA-RH7777 orthotopic liver tumors treated by microtripsy (i.e., intrinsic threshold CH, with 50–75% of the tumor volume treated) had tumor-free survival to the 3-month study endpoint, whereas 11 controlled untreated rats had to be sacrificed at 2–3 weeks after tumor inoculation due to the fast spread of liver metastases (Figure 4*b*).

Beyond the ability of histotripsy to destroy targeted tumor tissues, preclinical studies have pointed to the possibility that local histotripsy tumor ablation may also yield systemic antitumor immune effects. CH tumor ablation of murine melanoma and hepatocellular carcinoma tumors induced CD8^+^ T cell infiltration and growth inhibition of both local, partially ablated tumors and distant, nonablated tumors (abscopal effect) (Figure 4*c*) (47). One potential mechanism underlying the immune effects of histotripsy may relate to the purely mechanical nature of its cytotoxicity, in which cellular and subcellular structures are disrupted without the use of potentially immunosuppressive heat or ionizing radiation (118). Indeed, CH causes far more release of immunogenically intact tumor antigen and intratumoral CD8^+^ T cell infiltration than does radiofrequency ablation or radiation (47). Another potential mechanism may be the ability of histotripsy to induce specifically immunostimulatory pathways of cancer cell death. Investigations of BH in experimental models of breast and renal cell carcinoma have observed local release of tumor necrosis factor alpha (TNFα), activation of TNFα signaling pathways and inflammatory cytokines, and intratumoral infiltration of CD8^+^ T cells (48, 72). These events are hallmarks of immunogenic cell death (ICD), programmed pathways in which dying cells extrude proinflammatory intracellular substances called damage-associated molecular patterns (DAMPs) that activate innate and immune cells capable of mounting immune responses against the contents of dying cells (119). Indeed, analyses of tumor microenvironmental changes following CH and BH confirm significant release of DAMPs and upregulation of inflammatory signaling pathways within ablated tumors (32, 47). Transcriptomic analyses indicate that CH tumor ablation triggers a specific pathway of ICD called necroptosis, in which cells form self-destructive cell membrane pores through which DAMPs and tumor antigens are released. The immunogenic nature of the resulting tumor microenvironment is underscored by experimental observations that tumor homogenates generated by CH (but not by freeze-thaw dissociation or radiation) can function as vaccines that protect naive mice from tumor challenge. The mechanistic effects of CH-induced ICD are further underscored by the repeatable sequence of antigen-presenting cell (APC) activation in the ablation zone, APC priming of T cells in tumor-draining lymph nodes, and systemic activation and trafficking of antitumor CD8^+^ T cells that follows histotripsy tumor ablation (120).

The ability of histotripsy to induce ICD and antitumor CD8^+^ T cell responses has motivated strong interest in the concept of using histotripsy to sensitize tumors to the effects of checkpoint inhibition immunotherapy. The combination of BH with CD40 ligation polarizes macrophages and T cells against tumors and, when used in conjunction with anti-CTLA-4 (anti–cytotoxic lymphocyte antigen 4) and anti-PD-L1 (anti–programmed cell death ligand 1) antibodies, enhances the antimelanoma efficacy of checkpoint inhibition immunotherapy (121). Similarly, the combination of BH with anti-CTLA-4 and anti-PD-1 (anti–programmed cell death 1) antibodies results in maximal tumor control, survival, and favorable profiles of intratumoral lymphocytic infiltration in a murine model of pancreatic adenocarcinoma (88). CH alone dramatically enhances the antitumor effects of checkpoint inhibition (47). Interestingly, CD8^+^ T cells that are activated by CH exhibit the ability to induce ferroptosis, a specific process of cell death to which cancer cells are uniquely susceptible (122). Ferroptosis is the same mechanism by which checkpoint inhibition immunotherapy–activated CD8^+^ T cells exert their cytotoxicity (120), which may explain the potency of histotripsy–checkpoint inhibition combinatorial strategies (123).

### Cardiac Applications

CH has been investigated for treatment of hyperplastic left heart syndrome (HLHS), in which patients are born with an underdeveloped (or even the absence of a) left ventricle. As part of the reconstructive surgery, a flow channel needs to be created between the left and right chambers of the heart via a perforation through the atrial septum in neonatal HLHS patients (124). In fact, the need for an HLHS application motivated the invention and the first publication of histotripsy in 2004 (7). Ultrasound imaging–guided shock scattering CH was used to successfully create a perforation through the atrial septum in an in vivo open canine heart (10) and a perforation through the ventricular septum in a neonatal pig through an intact chest (26, 43). The short pulse duration (three cycles at 750 kHz–1 MHz) at a pulse repetition frequency (PRF) of 1–20 kHz and a peak negative pressure of 11–15 MPa are efficient at creating perforations through the cardiac tissue (7).

There are technical and safety issues specific for cardiac applications. Acoustic access to the heart from external applications is challenging due to the rib cage and lung blockage. However, even with ribs and a small part of the lung in the pathway, CH was successfully applied externally from the subcostal and transthoracic window (43, 44). Heart motion is another technical challenge when treating a cardiac target. Once cavitation is initiated, it tends to regenerate at the same site when the PRF is fast enough, leveraging the residual cavitation bubble fragments from the previous ultrasound pulse. This cavitation memory effect was utilized to apply accurate treatment in the presence of cardiac motion without actively applying any tracking correction (43, 44). Lastly, there is a concern that histotripsy may release tissue debris to cause hazardous emboli. MRI and histology of the brain and lung after CH cardiac treatment did not show any evidence of embolization (26, 43). The tissue debris induced by CH in vitro was measured to be smaller than 6 μm in more than 99% of the debris particles, with the largest particle measured at 54 μm (125).

### Blood Clots

The formation of pathological blood clots is the cause of many diseases. Histotripsy has been investigated to treat deep vein thrombosis (DVT) by mechanically liquefying and removing pathological blood clots. First, CH was employed to remove a blood clot in a vessel with the first indication of DVT. Shock scattering CH liquefied the target clot in an in vitro flow model (126) and in an in vivo porcine DVT model (33). Treatments were applied using five-cycle pulses at a PRF of 1 kHz and a peak negative pressure of 14–19 MPa with a 1-MHz focused transducer. In the in vivo DVT model, a clot that had formed in the femoral vein in the leg was treated. The vessel structure remained intact after CH, but the endothelial layer was damaged, and some hemorrhage was observed in the vessel wall. This occurs because the length of the cavitation cloud in shock scattering CH sometimes exceeds the width of the small vessel lumen of the femoral vein (<4–6 mm).

**Figure 5:**
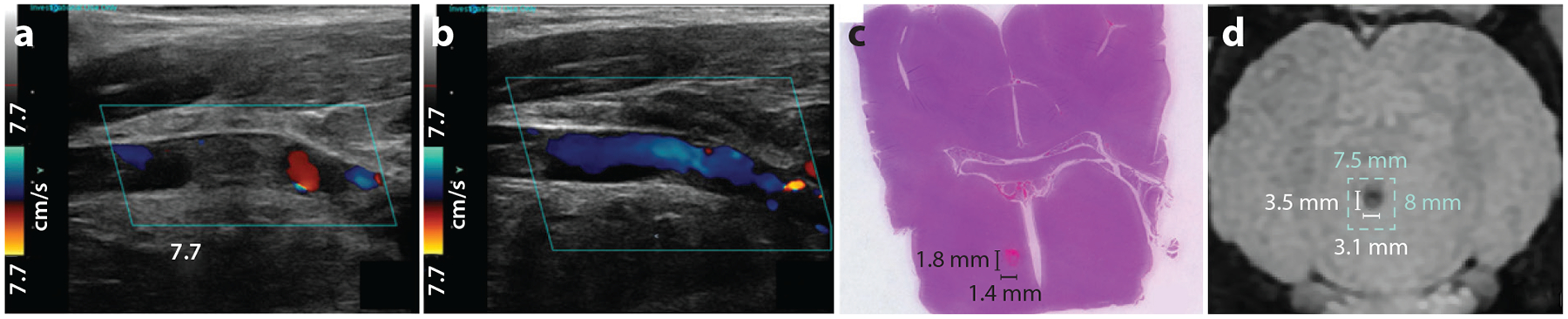
Histotripsy for preclinical blood clot and brain applications. (*a*,*b*) Color Doppler ultrasound images of an in vivo porcine femoral vein with deep vein thrombosis before and after microtripsy (34). (*a*) The blood clot occluded the vein before treatment. (*b*) The clot was removed and the vein recanalized by microtripsy after treatment. (*c*) Hematoxylin and eosin stained slide and (*d*) T1-weighted magnetic resonance (MR) image of an in vivo pig brain treated by MR-guided histotripsy through an excised human skull (29).

To avoid damage to the vessel wall, microtripsy was applied using one-cycle pulses at a PRF of 5–100 Hz and a peak negative pressure of 30–36 MPa by a 1-MHz focused transducer (127). Microtripsy achieved thrombolysis in in vitro (127) and in vivo porcine DVT models (34). In the in vivo treatment, flow was restored in the occluded vessel as confirmed by color Doppler ultrasound imaging (Figure 5*a*,*b*), and no vessel damage was observed 2 weeks after the treatment. Microtripsy was also demonstrated to be effective in treating retracted clots, although the treatment speed was slower for retracted clots compared with acute clots (128). The clot debris particles were measured to be smaller than 100 μm, with >96% of the volume smaller than 5 μm in diameter, which is sufficiently small to avoid hazardous emboli. Cavitation generated in the vessel formed a vortex flow to trap and fractionate any large particles to avoid embolization (129).

CH can be combined with thrombolytic drugs (e.g., recombinant tissue plasminogen activator) to enhance the treatment efficacy of thrombolysis (130, 131). CH-induced cavitation can mechanically create pores in the clot to facilitate permeation of the thrombolytic drugs into the clot. This is particularly helpful for treatment of retracted clots, which have dense fibrin networks and low porosity (132).

Histotripsy was also used to liquefy large hematomas resulting from trauma or postsurgical bleeds to facilitate fine-needle aspiration of the resulting fluid. These hematomas exert pressure on surrounding tissues, causing pain and affecting circulation, which may lead to organ failure. Unlike intravascular clots, large hematomas do not become noticeably stiffer with retraction, and they slowly liquefy over a protracted period of time (133). Thus, complete liquefaction of a hematoma volume is not required, as long as it is reduced enough to alleviate the pressure on surrounding tissues. BH, shock scattering CH, and HH regimes rapidly liquefy large hematoma volumes in vitro, which can then be aspirated with a needle (45, 133). The fastest volumetric liquefaction rate of 2.6 mL/min was achieved with HH and continuous focus translation, followed by BH and shock scattering CH. In an alternative approach, a needle or a catheter sheath was introduced into the hematoma closely adjacent to the focus and the liquid was aspirated continuously from the forming cavity, thus bringing the untreated clot volume into focus without needing to move the transducer. For BH, this approach demonstrated liquefaction rates of up to 5 mL/min in vitro and 3.3 mL/min in vivo in a porcine model of intramuscular hematoma (45).

### Brain Applications

Transcranial brain treatment is probably the most technically challenging application for histotripsy, because it requires very high pressure while the skull attenuates 70–90% of the acoustic power. However, transcranial histotripsy brain treatment also presents new opportunities. Transcranial MR-guided focused ultrasound thermal ablation is limited to treating a small volume in a central location in the brain due to overheating of the skull. As histotripsy mechanically breaks down the target tissue, if sufficiently high pressure can be achieved to perform transcranial histotripsy in the brain, a low duty cycle (e.g., <0.1%) can be used to mitigate skull heating and allow treatment of a wide location and large volumes inside the brain.

To date, three transcranial CH array transducers have been designed and constructed to generate >20-MPa peak negative pressure through an ex vivo human skull. All three arrays are hemispherical with a focal distance of 15 cm, including a 256-element, 250-kHz array; a 256-element, 500-kHz array; and a 360-element, 700-kHz array (76, 134). In addition, less-focused, fully populated phased arrays of 1-MHz frequency have recently been designed for transcranial BH applications (135).

Preclinical studies in the brain have been done using only CH so far. Microtripsy parameters were used to ensure the highest treatment accuracy: one-cycle pulses, >30-MPa peak negative pressure, and <0.1% duty cycle to mitigate skull heating (134). Microtripsy was demonstrated to treat a blood clot or a tissue phantom through an excised human skull in both deep locations and shallow locations (as close as 5 mm to the skull surface), as well as treating a volume of ex vivo brain tissue (e.g., 30 mL) while keeping the skull temperature increase below 4°C (134). The initial safety of CH brain treatment was shown in an in vivo porcine brain, where no excessive bleeding, edema, or other significant adverse events were observed (30). A transcranial 700-kHz MR-guided CH system was used to successfully ablate an in vivo porcine brain through an excised human skull (29) (Figure 5*c*,*d*). These results suggest the potential of using transcranial histotripsy to perform noninvasive ablation in the brain for treatment of brain tumors in various locations and volumes.

Transcranial histotripsy was also investigated to treat intracerebral hemorrhage (ICH) by liquefying a clot and aspirating it with a catheter. Using 250-kHz and 500-kHz arrays with microtripsy parameters (one-cycle pulses, 200-Hz PRF, and peak negative pressure of >30 MPa), a 4-cm-diameter spherical ex vivo clot was liquefied through an excised human skull at a rate of up to 16 mL/min (134). Shock scattering CH and BH were also demonstrated to liquefy large clots for ICH treatment, but the treatment was not done through the skull (46). The safety of microtripsy ICH treatments was examined in an in vivo porcine ICH model (49), where clots formed in pig brain were liquefied by microtripsy and aspirated with a needle. The pigs survived for a week without any clinical or neurological adverse effects.

The main technical challenges for transcranial histotripsy are skull-induced aberration and attenuation. The speed of sound in bone is much higher than that in soft tissue; thus, variations in skull thickness and density result in significantly reduced focal pressure and a distorted focal field when ultrasound propagates through the skull, an effect termed aberration. To correct skull-induced aberration, analytic computation based on CT head scans has been performed (136) to correct for focal shifts due to aberration (76, 82), but the efficacy of using this approach for focal pressure recovery is very limited. A two-step aberration correction was developed using a transmit-and-receive-capable transcranial histotripsy array (137) for more effective aberration correction, with the first step being a CT-based analytic correction approach and the second step being a cavitation-based correction approach (82). The shock-wave signal emitted from the cavitation received by each histotripsy array element is used to calculate the ultrasound travel time between the cavitation focus and each element. The travel time differences are then compensated at the ultrasound transmission to correct the aberration. Two-step aberration correction has shown close to perfect focal pressure recovery as compared with the use of direct acoustic hydrophone measurement at the focus (82, 135).

MRI is the clinical standard tool for diagnosis and therapy follow-up of brain diseases. MRI can be used for pretargeting, treatment monitoring, and posttreatment evaluation of transcranial histotripsy brain treatment, as described above in the section titled Image-Guided Targeting and Monitoring, but it requires an MR-compatible transcranial histotripsy array and specialized MRI pulse sequences. Alternatively, acoustic monitoring of cavitation is possible through the skull. Transmit-and-receive transcranial histotripsy can be used to receive acoustic cavitation emission signals to monitor and map cavitation (83). With such acoustic cavitation monitoring capability, transcranial histotripsy could be performed outside the MRI scanner and be guided by a neuronavigation system.

### Other Applications

In addition to cancer, cardiovascular, and neurological applications, many other applications have been investigated for preclinical histotripsy studies, as described in the following subsections.

#### Bactericidal effects.

Histotripsy is capable of fractionating not only cells but also bacteria that are much smaller (submicrometer) and more resilient to physical impacts (138). Histotripsy was applied to bacterial inactivation in two distinct contexts—clearing biofilms from the surfaces of catheters or implants and disinfection of abscesses (39, 138–140). A biofilm comprises a protective polysaccharide matrix layer adherent to a surface and up to tens of micrometers thick, within which one or more bacterial species are embedded. Biofilms can form on the surfaces of implanted materials from planktonic bacteria introduced externally and are very resistant to systemic antibiotic treatments. Shock scattering and intrinsic threshold CH regimes successfully eliminated biofilms harboring *Staphylococcus aureus* and *Pseudomonas aeruginosa* from the surfaces of surgical meshes and ureteral catheters, respectively, without affecting the integrity or strength of the surfaces. An average of 3.8-log kill was reported over surgical meshes (139), with some bacteria likely directly fractionated and some dislodged and washed off into the adjacent fluid, where the protective effect of biofilm is no longer present. Similarly, the average inactivation of *P. aeruginosa* in suspension within a catheter was 3.45 log (140).

Abscesses are collections of pus and bacteria ranging from liquid to cheeselike consistency and walled off from surrounding tissue by a fibrous capsule. Similar to biofilms, abscesses are resistant to systemically administered antibiotics and are typically treated with surgical incision and drainage (superficial) or percutaneous drain catheter placement (deep), both of which are associated with complications and discomfort. BH and shock scattering CH both liquefied an abscess to facilitate drainage and inactivated bacteria within the abscess in bimicrobial (*Escherichia coli* and *Bacteroides fragilis*) porcine abscesses in vivo. BH was more efficient at liquefaction, and CH was more efficient at disinfection (39). Thus, a treatment strategy combining the two regimes—liquefaction followed by disinfection—was deemed the most promising and yielded a 3.3-log kill (39).

#### Biofabrication, tissue decellularization, and sample prep for DNA analysis.

Tissue selectivity of histotripsy—the ability to fractionate cells and leave fibrous extracellular matrix and tissue structures intact—has potential uses in regenerative medicine. For example, Pahk et al. (141) showed an area of liver that was fractionated by BH in a mutant rat model deficient in albumin production. Extracellular matrix, biliary structure, and vascular network within the cavity remained intact and patent and facilitated the delivery of systemically injected donor hepatocytes that replaced dysfunctional liver tissue and led to albumin production. Alternatively, large tissue volumes could be decellularized by histotripsy ex vivo to form biocompatible scaffolds, as shown in proof-of-concept BH (20). This procedure was recently performed in artificially perfused porcine liver, demonstrating that BH of the liver allowed isolation and culture of hepatocytes with a high rate of viability after 1 week in culture (142). Another interesting in vitro application is accelerated preparation of samples—high-yield DNA extraction and chromatin fragmentation—for downstream genetic and epigenetic analyses (138, 143).

#### Histotripsy-facilitated liquid biopsy.

Liquid biopsy refers to sampling of blood-based biomarkers of certain tissue from the circulation. Of particular interest are cell-free circulating nucleic acids—microRNAs (miRNAs), messenger RNA, and DNA—that are very tissue specific and frequently dysregulated in many conditions, including cancer. However, two key challenges associated with detection of these biomarkers are their low quantities in the blood and the lack of identification of their anatomic origin. It was thus hypothesized that liquefaction of a very small area of tissue with histotripsy could release these (mostly intracellular) nucleic acid biomarkers into the extracellular space and into the circulation, increasing the sensitivity of blood-based sampling (52). This concept has been demonstrated in a rat prostate cancer model, wherein partial BH of a subcutaneous tumor resulted in immediate and transient elevation of circulating tumor-specific miRNAs but not broadly expressed miRNAs. A similar approach using milder cavitation-based stimulus was recently demonstrated to enhance the release of tumor DNA from a murine and porcine model of glioblastoma (144). This cavitation-enhanced release of cell-free nucleic acids can be used as a diagnostic tool, for anatomically resolved biopsy of sites that are difficult to sample with a needle, or as a therapy guidance tool to obtain information on the genetic content of a tumor during its ablation by histotripsy. This could inform the choice of combination treatments with chemotherapy or immunotherapy.

#### Histotripsy erosion of solid targets: kidney stones, bone tumors, and calcified aortic stenosis.

Similar to its application in soft tissues, histotripsy is capable of eroding solid, calcified targets down to micrometer-sized debris. Model kidney stones were the first such targets eroded with shock scattering CH (145). While the erosion rate was slower than that achieved with a clinical piezoelectric lithotripter, the size of the fragments did not exceed 100 μm, in contrast with millimeter-sized fragments by lithotripsy that can be difficult to pass. Another challenging target is bone tumors, which can have a heterogeneous composition including a soft tissue component and a lytic bone tumor component that consists of an osteoid matrix with tumor cells within. Intrinsic threshold CH was successfully used to ablate ex vivo and in vivo canine osteosarcomas (146). As expected, the ablation rate was substantially slower than is typical for soft tissue fractionation (approximately 0.25 mL/h) but resulted in the disintegration of the osteoid matrix (where present) into smaller fragments and complete lysing of the embedded tumor cells. Interestingly, the same treatment parameters did not have any effect on healthy canine cortical bone and sciatic nerve. Fragmentation of calcifications within the aortic valve leaflets with shock scattering CH in the context of calcified aortic stenosis (CAS) is the third example of histotripsy erosion (147). In ex vivo and in vivo porcine studies, the cavitation bubble cloud was confined within the thickness of the calcified leaflet, and after an hour of treatment the leaflet was softened by fragmenting the calcification without disrupting the superficial structures of the leaflet. The CAS application has undergone the first in-human trial, discussed below.

### Clinical Trials

To date, all human trials have been conducted using shock scattering CH. Two phase I human trials have been completed and one multicenter trial is ongoing, performed using CH devices manufactured by HistoSonics, Inc. (Plymouth, Minnesota and Ann Arbor, Michigan, USA). The first histotripsy trial [phase I, National Clinical Trial (NCT) number NCT01896973] was conducted in two clinical centers in the United States in patients with benign prostatic hyperplasia (BPH) in 2016–2017. BPH is characterized by an enlarged prostate gland compressing the urethra and causing uncomfortable urinary symptoms, such as blocking the flow of urine out of the bladder. CH was used to liquefy the in vivo canine prostate tissue surrounding the urethra, which would be urinated out. Within 28 days, the destroyed urethra was reendothelialized to form a larger urination channel. In human, CH was applied from the perineal position and guided by transrectal ultrasound imaging. In all patients, cavitation was observed on ultrasound imaging at the target location. All subjects received 60 min of acoustic energy delivery. No serious adverse events were observed. Improvements in the International Prostate Symptom Score and other symptom scores exceeded what would be expected for a placebo response. However, in contrast with the results from canine studies, no tissue debulking effect was seen with ultrasound or endoscopic evaluation. It was later found that human prostate is much more fibrous than canine prostate and thus more resistant to histotripsy (115). It is likely that not enough of a CH dose (number of pulses) was delivered in the human BPH trial to homogenize the prostate tissue. Recent BH studies in human prostate autopsy samples showed complete liquefaction of prostate tissue and tumors, including BPH and adenocarcinoma (87, 115).

**Figure 6:**
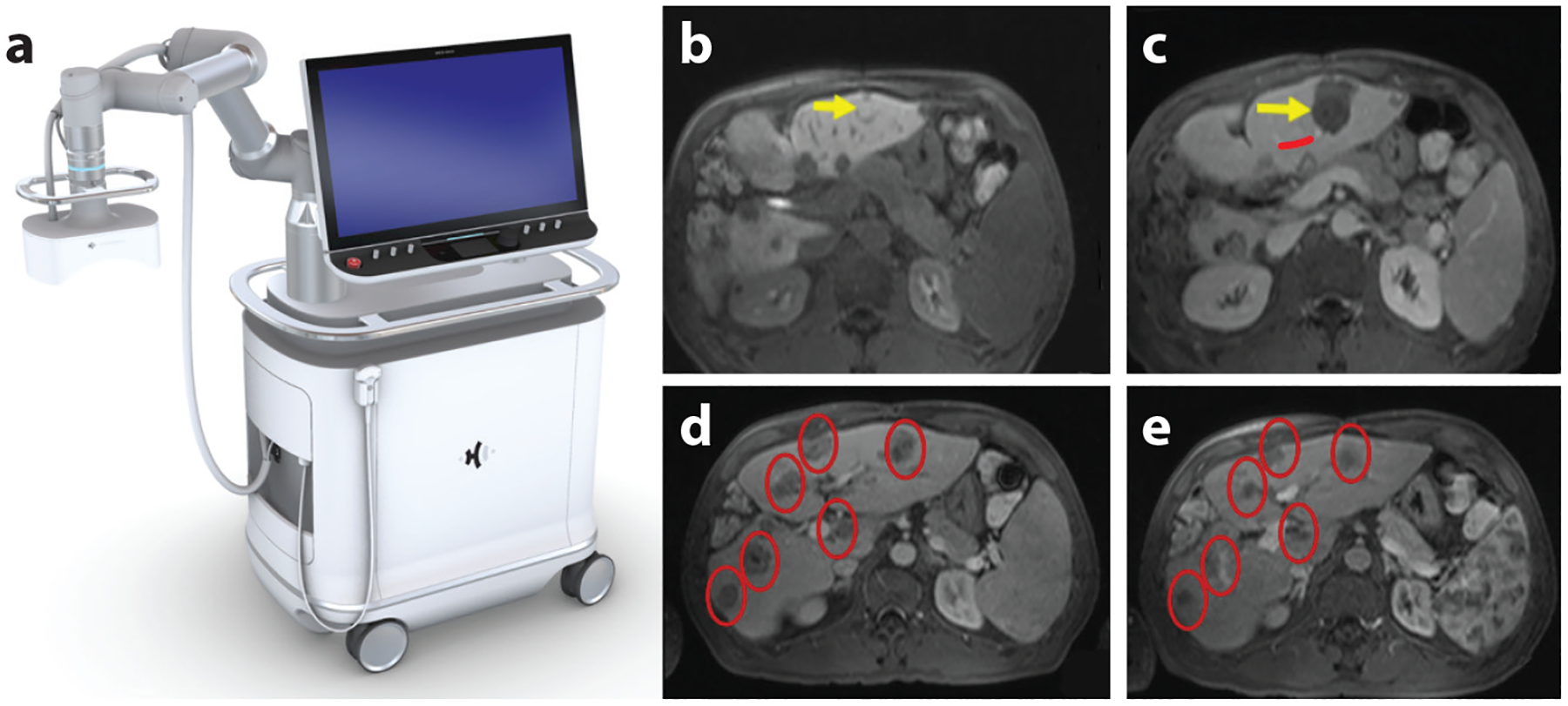
Results from clinical trials. (*a*) An image of the ultrasound image–guided clinical prototype histotripsy system (Edison^™^ platform; HistoSonics, Inc.). (*b*–*e*) Magnetic resonance (MR) images from the THERESA histotripsy liver tumor clinical trial (51). (*b*,*c*) MR images of the targeted tumor 5 mm adjacent to the hepatic vessel branch (*b*) before and (*c*) after shock scattering cavitational histotripsy, showing effective ablation of the liver tumor (tumor indicated by the *yellow arrow* in panel *b*) with a margin (ablation indicated by the *yellow arrow* in panel *c*) and intact hepatic vessel (indicated by the *red line* in panel *c*). (*d*,*e*) MR images of nontargeted tumors (*red ovals*) (*d*) before and (*e*) 8 weeks after histotripsy treatment, showing the abscopal response as a size reduction.

In 2019, a phase I trial (THERESA, NCT03741088) was conducted in patients with liver tumors in Barcelona, Spain (9). All patients had noncurable multifocal liver malignancy. Histotripsy was delivered to treat 11 tumors (all <3 cm in diameter) in eight patients using the Edison^™^ platform (HistoSonics) (Figure 6*a*), with a 750-kHz focused ultrasound transducer. There was one mistargeting, because the tumor could not be visualized clearly on ultrasound. For 10 of 11 tumors, the entire tumor with an ~5-mm margin was successfully targeted and ablated, as confirmed by MRI (Figure 6*b*,*c*). The treatment time was proportional to the ablation volume and was ~30 min for a spherical volume of 3 cm in diameter. Nine of the 10 treated tumors had local tumor regression observed at a 2-month follow-up (average 72% volume retraction), while 1 tumor got connected to the adjacent tumor, so its growth could not be accurately measured. No major adverse events were observed. Interestingly, in two patients, there was a continuous decline in tumor markers, and substantial off-target tumor shrinkage was observed on MR images in one patient (Figure 6*d*,*e*) (51). This is the first human trial of histotripsy treatment of malignant tumors. The results not only demonstrated the safety of CH treatment of liver tumors but also showed its promise to achieve local tumor regression and potential abscopal effects.

The enrollment of a multicenter clinical trial (HOPE4LIVER) on CH treatment of liver tumors was completed in the United States (eight sites; NCT04572633) and Europe (six sites; NCT04573881) in 2023 using the Edison platform (HistoSonics), and the results are expected to release shortly. Based on the HOPE4LIVER trial results, the Edison platform was recently approved by the FDA for histotripsy treatment of liver tumors.

The third CH phase I human trial was conducted on patients with CAS (NCT03779620) in France and the Netherlands in 2019 (148, 149). Ten patients with severe calcific aortic valves were treated using the Valvosoft transducer device (Cardiawave, France), guided by ultrasound imaging. Traditional shock scattering CH parameters were used: five cycle pulses at a peak negative pressure of 15–20 MPa, PRF of 100–300 Hz, a 0.25% duty cycle by an external multielement focused ultrasound transducer (bandwidth of 700 kHz–1.25 MHz), and a total of 60 min of treatment. Cavitation was generated and observed by ultrasound imaging. Of the 10 patients, 6 had an increased softened valve area and were classified as responders, and 4 were classified as nonresponders (149). No patients experienced significant adverse effects. In contrast to soft tissue histotripsy, where tissue homogenization was produced, cavitation was used to soften the calcified regions of the aortic valve.

## FUTURE OF THE FIELD

The noninvasive, nonionizing, and nonthermal nature of histotripsy is expected to allow it to improve treatment, especially for patients who are not suitable candidates for surgery. The recent FDA approval of the ultrasound image–guided Edison platform (HistoSonics) for histotripsy ablation of liver tumor opens up the clinical use of this new technology. The Edison platform can also be used to treat other abdominal organs (e.g., kidney, pancreas) but would require additional clinical trials. So far, there have been histotripsy clinical trials for three indications (BPH, liver tumors, and CAS), with a new clinical trial started on renal tumors (NCT05432232) in 2023. However, histotripsy is a platform technology and can be expanded to treat many organs and different diseases, as described in the preclinical studies mentioned above.

Noninvasive HIFU thermal ablation has been in clinical use for many years, but it is limited by the high cost of MRI, slow treatment speed, and restricted treatment locations. With its tissue-selective ablation capability, histotripsy can be used to treat high-risk locations near critical structures to preserve these structures (e.g., major vessels, nerves, bile ducts, and connecting systems), which would be very difficult to access with surgical or current thermal ablation techniques, including HIFU thermal ablation. In comparison to the use of MRI guidance for HIFU, histotripsy uses ultrasound imaging for guidance, which has lower cost and easier access.

The treatment speed of the HistoSonics Edison platform is currently ~1 mL/min, which is on par with clinical radiofrequency ablation and faster than most HIFU systems. Edison currently uses a single focused ultrasound transducer and is mechanically moved by a robotic arm. Histotripsy treatment speed may be further increased using a phased array and electronic focal steering.

Histotripsy treatment locations are somewhat limited due to the ultrasound physics. As ultrasound propagation is blocked by bone and gassy organs, certain locations may not be accessible with external ultrasound delivery, for example, locations right behind the ribs or lungs. Histotripsy can still be created through bone blockage (e.g., rib cage or skull), if sufficiently high focal pressure can be achieved, as described in the abovementioned cardiac and brain applications. The aberration caused by bone blockage may be mitigated by conducting aberration correction (137). If the bowel is in the pathway (as in the case of pancreas, liver, or spleen), fasting can help remove air bubbles in the bowel to enhance acoustic propagation. It is also possible to perform hydrodissection by injecting fluid to displace nearby critical structures and create a protective barrier between those structures and the target region, which is a technique used during thermal ablation. Therefore, it is possible to expand histotripsy treatment locations using aberration correction and minimally invasive clinical procedure such as hydrodissection.

Histotripsy immunostimulation has attracted significant attention from both the clinical community and patients. The promising abscopal effect induced by histotripsy alone from preclinical studies and the THERESA human trial indicates the potential of using histotripsy to enhance immunotherapy to treat off-target tumors. The immunostimulation effect observed in the treatment of nonimmunogenic cancers such as liver cancer is particularly encouraging and is drawing many researchers to further study this topic. It is expected that there will be a major push to initiate a clinical trial on histotripsy-enhanced immunotherapy in the near future, which will help accelerate the widespread use of histotripsy, if it is successful.

## Figures and Tables

**Table 1:** Representative parameters of various histotripsy approaches

	CH		HH	BH
	Intrinsic threshold CH (microtripsy)	Shock scattering CH		
**Frequency**	0.25–6 MHz	0.25–5 MHz	1–5 MHz	1–5 MHz
**Pulse length**	1–2 cycles (0.2–4 μs)	3–20 cycles (0.6–80 μs)	0.2–1 ms	1–20 ms
**Duty cycle**	<1%	<1%	1%–2%	1%–2%
**Peak power**	>2,000 W	>1,000 W	>800 W	>300 W
**p− pressure**	>30 MPa	20–30 MPa	15–20 MPa	10–15 MPa
**p+/As pressure**	No requirement	>100 MPa	>80 MPa	>60 MPa
**Lesion width/length (at 1 MHz)**	0.3–1 mm/0.5–2 mm	1 mm/4 mm	2 mm/5 mm	4 mm/8 mm

Abbreviations: BH, boiling histotripsy; CH, cavitational histotripsy; HH, hybrid histotripsy.
